# Validity and reliability of the Korean version of the Örebro Musculoskeletal Pain Screening Questionnaire

**DOI:** 10.1097/MD.0000000000049753

**Published:** 2026-07-10

**Authors:** Kyoungrim Kang, Dong-Chul Moon, Sang-Hwa Lee, Kyo-Yeon Park

**Affiliations:** a College of Nursing, Research Institute of Nursing Science, Pusan National University, Yangsan-si, Gyengsangnam-do, Republic of Korea; b Department of Physical Therapy, Gimhae University, Gimhae-si, Gyeongsangnam-do, Republic of Korea; c Department of Nursing, Gimhae University, Gimhae-si, Gyeongsangnam-do, Republic of Korea; d College of Nursing, Pusan National University, Yangsan-si, Gyengsangnam-do, Republic of Korea.

**Keywords:** confirmatory factor analysis, low back pain, musculoskeletal, screening tool, Örebro Musculoskeletal Pain Screening Questionnaire

## Abstract

Low back pain (LBP) is a major musculoskeletal cause of functional disability worldwide. Psychosocial factors are essential in its progression; however, the assessment of psychosocial dimensions in non-Western contexts, including Korea, remains understudied. The Örebro Musculoskeletal Pain Screening Questionnaire (OMPSQ) is widely used to evaluate psychosocial risk factors associated with chronic pain, yet its Korean version has not been fully validated. This study aimed to translate the OMPSQ into Korean and examine its psychometric properties to confirm its suitability for assessing psychosocial factors in Korean patients with LBP. This methodological study followed standardized guidelines for cross-cultural adaptation, including forward–backward translation and expert panel review. Ethical approval was obtained, and 163 adult patients with LBP were recruited from 4 physiotherapy clinics in South Korea. Confirmatory factor analysis was conducted to assess construct validity, while concurrent validity was examined using correlations with the numerical rating scale, the STarT Back Screening Tool (SBST), and the Korean version of the Fear-Avoidance Beliefs Questionnaire. Internal consistency was assessed using Cronbach α. Confirmatory factor analysis of the Korean OMPSQ demonstrated that the model fit indices were acceptable (χ^2^ = 282.44, root mean square error approximation  = 0.06, comparative fit index = 0.92, goodness-of-fit index  = 0.86). Concurrent validity and reliability were supported by significant correlations between the OMPSQ subscales and related instruments, including numerical rating scale, STarT Back Screening Tool, and Korean version of the Fear-Avoidance Beliefs Questionnaire (correlation coefficients: 0.50–0.62, *P* < .001) and high internal consistency (Cronbach α = 0.84). The Korean version of the OMPSQ showed satisfactory validity and reliability, retaining the original 5-factor structure, and may be used to assess psychosocial factors in Korean patients with LBP in primary physiotherapy contexts within South Korea. Further studies are needed to extend its validation across diverse cultural and clinical settings.

## 1. Introduction

Low back pain (LBP) is a common condition experienced by 84% of the population at least once during their lifetime. It is one of the major musculoskeletal disorders that causes functional impairment and increases social and economic costs.^[1,2]^ The average lifetime prevalence of LBP is 39%, with a point prevalence of 18% and a 1-month prevalence of 31%.^[3]^ In particular, 85% to 95% of patients with LBP have nonspecific low back pain (NSLBP), for which no clear pathological cause has been identified,^[4]^ many of whom experience recurrent episodes, and the majority suffer from chronic pain.^[5]^ Given the high prevalence and recurrent nature of NSLBP, a purely biomedical explanation is often insufficient to account for symptom persistence and disability.^[6]^ Contemporary frameworks, including the World Health Organization’s conceptualization of chronic pain, emphasize dynamic interactions among biological, psychological, and social factors, indicating that unaddressed psychosocial factors may contribute to recurrence and prolonged disability and underscoring the importance of early psychosocial screening in NSLBP.^[7,8]^

In the process of LBP becoming chronic, not only physical and structural issues but also psychosocial factors such as kinesiophobia, fear-avoidance beliefs, and pain catastrophizing are significant factors. Approximately 53% of patients with chronic LBP experience accompanying psychological disorders.^[9,10]^ These psychosocial factors should be considered when assessing and treating LBP. However, despite the recognized importance of psychosocial factors in the chronicity of LBP, their systematic assessment in routine clinical practice remains complex and challenging, highlighting the need for brief and clinically feasible instruments to support prognostic stratification and clinician decision-making in patients with NSLBP.^[11–13]^

The Örebro Musculoskeletal Pain Screening Questionnaire (OMPSQ) is a brief self-assessment tool developed by Linton and Hallden^[14]^ to screen patients with LBP for psychosocial factors and associated risk of developing chronic pain and disability. It contains 25 items organized into 5 domains: pain, function, psychological, fear-avoidance, and miscellaneous. The OMPSQ is designed in a simple questionnaire format, making it easy for patients to self-complete and allowing for rapid assessment in a clinical setting. With a high reliability of 0.83 and moderate predictive validity of 0.68 to 0.83,^[14,15]^ the OMPSQ is a valuable tool for assessing psychosocial factors in patients with LBP.

Commonly used instruments for assessing patients with LBP differ substantially in their domain coverage and intended clinical purpose. The StarT Back Screening Tool (SBST) provides brief risk stratification by incorporating both physical and psychosocial items, primarily to guide stratified treatment pathways rather than to comprehensively assess prognostic factors.^[16]^ The Fear-Avoidance Beliefs Questionnaire (FABQ) focuses specifically on fear-avoidance beliefs related to physical activity and work, thereby targeting a single psychological construct.^[17]^ The numeric rating scale (NRS) is a simple and widely used tool that quantifies pain intensity only and does not capture functional or psychosocial dimensions.^[18]^ In comparison, the OMPSQ provides an integrated assessment of multiple prognostic dimensions within a single brief instrument, supporting multidimensional psychosocial risk screening in routine clinical practice.^[19]^

Currently, the OMPSQ has been translated into several languages in addition to English, including Norwegian, Spanish, French, Brazilian, and Chinese. It has demonstrated reliability and validity as a predictor of long-term disability and work absenteeism associated with psychosocial yellow flags in patients with musculoskeletal pain.^[20–24]^ However, the OMPSQ has not yet been translated into Korean, and there is a lack of research on its use. Because psychosocial questionnaire items may be sensitive to linguistic nuance and sociocultural context, cross-cultural adaptation is recommended to ensure conceptual equivalence rather than literal translation.^[25]^ In Korea, stigma related to mental illness may reduce willingness to endorse distress-related questionnaire items, potentially influencing responses to psychosocial screening measures.^[26]^ In addition, distress in Korean cultural contexts may be communicated through somatic language, which could affect how psychologically oriented items are interpreted and answered.^[27]^ Therefore, this study aimed to translate the OMPSQ to suit the Korean culture and clinical environment while maintaining the uniqueness of the original instrument. It also aimed to validate the validity and reliability of the Korean version of the OMPSQ to provide a basis for assessing psychosocial factors in patients with LBP. We hypothesized a priori that the Korean OMPSQ would retain the original 5-domain structure with acceptable confirmatory factor analytic model fit; demonstrate concurrent validity through moderate correlations in the expected direction with related measures (NRS, SBST, and FABQ-K); and show acceptable internal consistency reliability for the total scale.

### 1.1. Study objective

This study aimed to translate the OMPSQ developed by Linton and Hallden^[14]^ into Korean and to test its validity and reliability in patients with LBP

## 2. Materials and methods

### 2.1. Study design

This methodological study aimed to translate the OMPSQ developed by Linton and Hallden^[14]^ into Korean and to verify the validity and reliability of the Korean version of the OMPSQ among Korean patients with LBP.

### 2.2. Study participants

The study participants were adult patients with LBP who visited 4 physiotherapy clinics in cities G, P, and S in South Korea. To enhance sample heterogeneity and mitigate potential urban bias, participants were recruited across multiple clinics serving different geographic areas. In accordance with Strengthening the Reporting of Observational Studies in Epidemiology reporting recommendations,^[28]^ the inclusion and exclusion criteria were defined as follows:

Inclusion criteria:

- Adults aged 18 years or older
- Diagnosis of NSLBP
- Ability to understand and complete self-report questionnaires

Exclusion criteria:

- Presence of red-flag pathologies (e.g., fracture, malignancy, and inflammatory spinal disease)
- Cognitive impairment or neurological conditions limiting questionnaire completion
- Ongoing litigation or compensation claims related to LBP

The sample size for this study was 150. This calculation was based on a previous study suggesting that the minimum sample size for assessing the validity and reliability of a measurement tool should be 5 to 7 times the number of items^[29]^ and that at least 100 participants are required for analysis.^[30]^ In accounting for a 10% dropout rate, 165 questionnaires were distributed to 165 participants. A total of 163 copies were used for the final analysis, excluding 2 copies that could not be processed because of nonresponse.

### 2.3. Instruments

This study used a 56-item self-report questionnaire consisting of a 5-item general characteristics questionnaire (excluding the 4-item general characteristics of the OMPSQ), a 1-item NRS, a 25-item OMPSQ, a 16-item FABQ-K, and a 9-item SBST.

#### 2.3.1. General characteristics

The general characteristics in this study consisted of 9 questions, including height and weight, education, history of LBP, and date of recent onset, and 4 general characteristics questions from the OMPSQ, which asked about the year of birth, sex, place of birth, and employment.

#### 2.3.2. Örebro Musculoskeletal Pain Screening Questionnaire

The OMPSQ is a self-assessment tool developed by Linton and Hallden^[14]^ to screen for psychosocial factors and risk of developing chronic pain and disability in patients with LBP. The tool comprises 25 items and includes 5 subscales: pain, function, psychological distress, fear-avoidance, and miscellaneous. Questions 1 to 4 ask about general demographic characteristics (year of birth, sex, place of birth, and employment status) and were not included in the scoring, consistent with the original OMPSQ. Scoring of the OMPSQ was conducted according to the original guidelines as follows: questions 1 to 4 were excluded from score calculation; question 5 was weighted by multiplying the response score by 2; questions 12, 16 to 17, and 21 to 25 were converted by subtracting each response score from 10; the remaining questions were scored on a 0 to 10 scale; and the total score was calculated by adding the converted and raw scores, which ranged from 0 to 210, with higher scores indicating higher levels of psychosocial risk and disability. Total scores of 105 or less were classified as low disability, 106 to 130 as moderate disability, and 131 or more as high disability.^[31]^ Previous studies have reported high internal consistency for the OMPSQ, with Cronbach α of 0.95 reported by Grotle et al^[20]^ and 0.88 by Chan et al^[32]^

#### 2.3.3. Numerical rating scale

The NRS was used to validate the concurrent validity of the OMPSQ subscales with pain. The NRS is a simple scale with 0 at the left end (no pain at all) and 10 at the right end (the most intense pain), which asks subjects to express their subjective pain intensity numerically. Higher scores indicate higher levels of LBP.

#### 2.3.4. STarT Back Screening Tool

The SBST, developed by Hill et al^[16]^ was used to assess the concurrent validity of the OMPSQ function and psychological subscales. It comprises 9 items, 3 on a 5-point scale and 6 on a 10-point scale. A total score of 3 or less was classified as low risk. A score of 4 or more but <3 on items 5 to 9 (psychosocial subscale) was classified as intermediate risk. Furthermore, a combined score of 4 or more and 3 or more on items 5 to 9 was classified as high risk. At the time of tool development, discriminant validity was reported to be areas under the curve= 0.70 (0.62–0.88), with Cronbach α of 0.87 in the Brazilian version^[33]^ and Cronbach a of 0.75 in the Japanese version.^[34]^ The reliability in this study was Cronbach α 0.92.

#### 2.3.5. Fear-Avoidance Belief Questionnaire-Korean

The FABQ-K, developed by Joo et al,^[35]^ was used to validate the concurrent validity of the OMPSQ’s fear-avoidance subscale. This instrument assesses fear-avoidant reactions and consists of 16 items and 2 subscales. The subscales were divided into 5 items for physical activity and 11 for work. Moreover, participants rated their level of agreement with each statement using a 5-point Likert scale. Five items (items 2, 8, 13, 14, and 16) were excluded from the total score, with a higher total score indicating a stronger fear-avoidance response. The instrument’s reliability was Cronbach α 0.95 when it was developed and Cronbach α 0.92 in this study.

### 2.4. Data collection and human participants’ protections

#### 2.4.1. Translation process of the tool

After obtaining permission to use the instrument from the original authors of the OMPSQ, Linton and Hallden,^[14]^ the translation process was conducted in accordance with the guidelines for cross-cultural adaptation.^[36]^

- Phase 1: Forward translation: The original English version of the OMPSQ was independently translated into Korean by 1 nursing major and 1 English literature major, who had never seen the instrument before and had extensive experience in translating between Korean and English. The 2 forward translations were then compared and discussed to resolve discrepancies.
- Phase 2: Back-translation: The reconciled Korean version was back-translated into English by a Korean–American bilingual with no prior knowledge of the instrument and a nursing professor with extensive experience in Korean and English translations. This phase aimed to check that the translated version was consistent in meaning and equivalent to the original version.
- Phase 3: Panel review and adjudication: An expert panel consisted of 5 members – 2 health professionals, 2 bilingual translators, and 1 researcher with expertise in cross-cultural measurement. The translations and back-translations were reviewed in the expert panel meeting to ensure consistency of scale terminology and correct wording that differed in meaning from the original. Discrepancies were resolved through discussion, and consensus was reached when agreement was achieved among the majority of panel members. The questions on general characteristics such as place of birth and type of employment were adapted to the local context, and expressions such as “upper back” and “lower back” were reworded to “back” and “waist” to reflect cultural appropriateness. Finally, the translated and back-translated versions were checked against the original to ensure no differences in meaning between the items, and the Korean version of the OMPSQ was finalized to reflect any additional cultural modifications.

### 2.5. Data collection

First, to collect data, recruitment documents detailing the purpose of the study and the methods and procedures for data collection were posted at the entrances of the 4 physiotherapy rooms located in G, P, and S. The URL of the online questionnaire (https://moaform.com/q/3Dt6kg) or QR codes were posted at the entrances of the 4 physiotherapy rooms. When the subjects read the recruitment documents and expressed their willingness to participate in the study, the 1st screen of the questionnaire was displayed, and the subjects were informed about the purpose and method of the study, participation criteria, questionnaire response method, participation reward, and data utilization. If they agreed to participate in the study, they were asked to click the “Take Survey” button to complete the online questionnaire so that they could participate in the study voluntarily. The recruitment document stated that the subjects could contact the physiotherapist or the researcher if they had any questions, and the research assistants were 4 physiotherapists at each hospital who had an understanding of LBP. Preliminary training was conducted via Zoom before data collection to help them answer any questions the subjects may have had. The questionnaire took approximately 20 minutes to complete, and the participants were offered a small gift as compensation for their participation.

### 2.6. Ethical considerations

This study was approved by the Institutional Review Board of Pusan National University (No. PNU_IRM/2023_56_HR) and was conducted in accordance with the principles of the Declaration of Helsinki.

- Informed consent and voluntariness: Potential participants were provided with detailed information regarding the purpose of the study, data collection procedures, eligibility criteria, questionnaire completion process, compensation, and data use through recruitment notices and the 1st screen of the online survey. Informed consent was obtained electronically prior to participation. Participants were informed that there would be no penalty for refusing to participate or withdrawing from the study at any time without penalty.
- Data protection and confidentiality: No personally identifiable information was collected during the survey process. The data collected would remain anonymous and never be used for any purpose other than the study. For the purpose of providing participation compensation, contact information was collected separately and stored on a password-protected personal computer accessible only to the principal investigator and co-investigators. After compensation distribution was completed, all contact information was immediately destroyed. Research data will be retained for 3 years following study completion and then permanently deleted.
- Participant protection: This study involved a self-administered questionnaire and was considered to pose minimal risk to participants. No sensitive items were included that were expected to cause significant discomfort. In the event of research-related distress, appropriate psychological support services would be provided at the expense of the research team.

### 2.7. Data analysis

Data were analyzed using SPSS for Windows software version 27.0 (IBM Corp., Armonk) and AMOS 26.0 (IBM Corp., Armonk). Participants’ general characteristics were described using frequencies, percentages, means, and standard deviations. Confirmatory factor analysis (CFA) was conducted to examine construct validity. Prior to factor analysis, the adequacy of the data was assessed using the Kaiser–Meyer–Olkin (KMO) and Bartlett tests of sphericity. Before performing CFA, the appropriateness of applying maximum likelihood estimation was examined by assessing the assumption of multivariate normality. To this end, skewness and kurtosis statistics of the observed variables were calculated to evaluate their distributional properties. This procedure was conducted as a preliminary step to ensure the validity of CFA using maximum likelihood estimation. In cases where violations of the normality assumption are identified, robust estimation methods such as bootstrapping may be considered as alternative analytical strategies. CFA was performed by fixing each item to its corresponding latent factor according to the subscale structure of the original instrument. The relationship between observed variables and latent constructs was estimated using the maximum likelihood estimate, which derives factor loadings based on the covariance matrix of the observed data. In the event of missing data, full information maximum likelihood was considered as the method for handling missing values under the assumption that data were missing at random, allowing cases to be retained in the analysis. Model fit was evaluated using multiple goodness-of-fit indices, including the chi-square (χ^2^) statistic, goodness-of-fit index (GFI), root mean square error of approximation (RMSEA), normed fit index (NFI), incremental fit index (IFI), Tucker–Lewis index (TLI), comparative fit index (CFI), parsimony normed fit index (PNFI), and parsimony comparative fit index (PCFI). Model fit was interpreted based on the criteria suggested by Hu and Bentler,^[37]^ whereby CFI and TLI values of 0.95 or higher and RMSEA values below 0.06 indicate a good model fit. To improve model fit, covariances between error terms within the same latent construct were added when the corresponding modification indices (MIs) exceeded 10. High MI values between error terms within the same factor were interpreted as indicating shared variance not sufficiently explained by the latent construct. Concurrent validity was examined using Pearson correlation coefficient to assess the relationships between the OMPSQ subscales and related instruments. The pain subscale of the OMPSQ was correlated with the NRS, the function and psychological subscales were correlated with the SBST, and the fear-avoidance subscale was correlated with the FABQ-K. Reliability was assessed by calculating Cronbach α to evaluate internal consistency.

## 3. Results

### 3.1. General characteristics

The study included 163 adult patients with LBP; their general characteristics are presented in Table 1. Overall, the sample consisted primarily of young to middle-aged adults who were predominantly engaged in paid employment, providing context for the psychosocial characteristics assessed in this study. General characteristics included age, sex, place of birth, height, weight, current employment status, level of education, experience of LBP, and duration of LBP. All participants were Korean (100%), with a mean age of 33.76 ± 10.21 years, and 53.4% (87 participants) were male. Regarding current employment, 77.9% (127 participants) were paid, followed by 9.2% (15 participants) who were unemployed. The highest level of education was university graduation. A total of 70.6% (115 participants) and 79.8% (130 participants) had a previous history of LBP, with a mean duration of 34.63 ± 52.01 months.

**Table 1:** General characteristics of participants (N = 163).

Variables	Categories	n (%) or M ± SD
Age (yr)		33.76 ± 10.21
Gender	Male	87 (53.4%)
	Female	76 (46.6%)
Place of birth	Korea	163 (100%)
	Outside Korea	0 (0%)
Height (cm)		168.98 ± 8.62
Weight (kg)		68.11 ± 16.61
Current employment	Paid employment	127 (77.9%)
	Academics	9 (5.5%)
	Unpaid work from home	0 (0%)
	Unemployed	15 (9.2%)
	Retirement	1 (0.6%)
	et al.	11 (6.7%)
Level of education	None	0 (0%)
	Graduated from primary school	0 (0%)
	Graduated from middle school	2 (1.2%)
	Graduated from high school	36 (22.1%)
	Graduated from university	115 (70.6%)
	Postgraduate and above	10 (6.1%)
Past experience of low back pain	Yes	130 (79.8%)
	No	33 (20.2%)
Duration of low back pain (mo)		34.63 ± 52.01

M = mean, SD = standard deviation.

### 3.2. Construct validity test

#### 3.2.1. Item and factor analysis

KMO and Bartlett test of sphericity were conducted to assess the suitability of the data for factor analysis for the 21 items, excluding the 4 general characteristic items. The KMO value was 0.83, indicating adequate sampling adequacy. Bartlett test of sphericity was statistically significant (χ^2^ = 1447.24, *P* < .001), confirming that the correlation matrix was appropriate for factor analysis.

As a preliminary analytic step, exploratory principal component analysis with varimax rotation was performed to examine the underlying factor structure and to confirm item suitability prior to CFA. Six factors with eigenvalues >1.0 were extracted. All 21 items, excluding the 4 general characteristic items, demonstrated factor loadings >0.40, satisfying the predefined criteria for item retention. Based on these results, all items were retained and entered into the CFA measurement model.

#### 3.2.2. Confirmatory factor analysis

**Figure 1.**
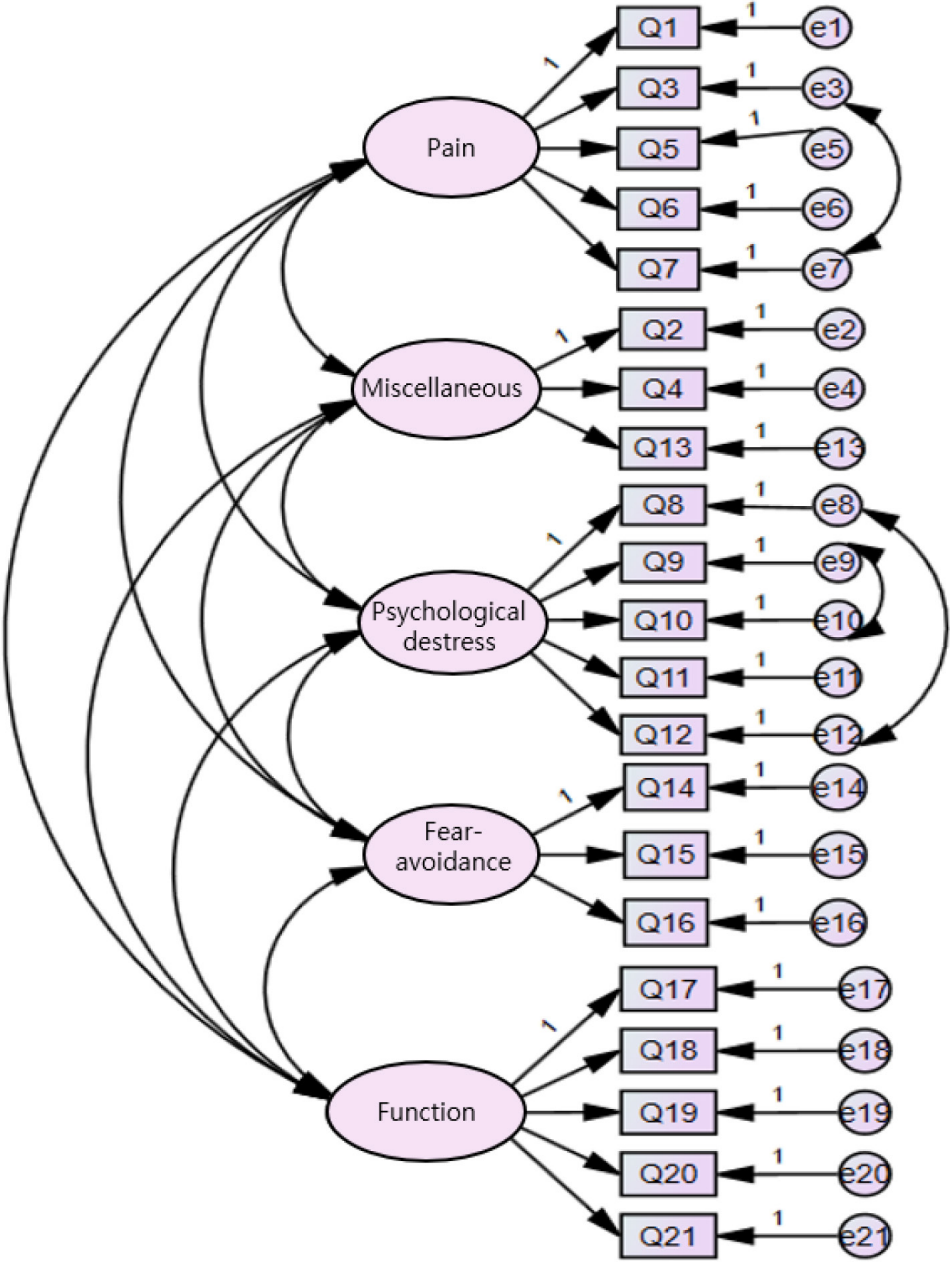
Confirmatory factor analysis of the OMPSQ-K. OMPSQ-K = Örebro Musculoskeletal Pain Screening Questionnaire-Korean.

CFA was subsequently conducted to examine whether the 21 items of the Korean version of the OMPSQ, excluding the 4 general characteristic items, were appropriately represented by the hypothesized 5-factor structure. The analysis was performed under the assumption of a 5-factor model derived a priori. Model fit was evaluated using multiple goodness-of-fit indices. According to Hair et al,^[38]^ RMSEA values between 0.05 and 0.10 indicate acceptable fit, while GFI and CFI values above 0.90 indicate good model fit. The initial CFA results yielded χ^2^ = 328.53 (*P*< 0.001), GFI = 0.84, RMSEA = 0.07, NFI = 0.79, IFI = 0.89, TLI = 0.87, CFI = 0.89, PNFI = 0.67, and PCFI = 0.76, suggesting that the initial model fit was not fully satisfactory. To improve model fit, MIs were examined. High MI values were identified between items 3 and 7 in the pain domain (MI = 17.615), items 9 and 10 in the psychological domain (MI = 10.740), and items 8 and 12 in the psychological domain (MI = 11.936). Accordingly, covariance between the corresponding error terms was added (Fig. 1).

The final CFA model demonstrated improved fit indices, with χ^2^ = 282.44 (*P* < 0.001), GFI = 0.86, RMSEA = 0.06, NFI = 0.82, IFI = 0.92, TLI = 0.90, CFI = 0.92, PNFI = 0.68, and PCFI = 0.77. Overall, the fit indices indicated an acceptable to good model fit, supporting the suitability of the 5-factor measurement model (Table 2). In the final CFA model, standardized factor loadings ranged from 0.30 to 0.92. While most items demonstrated acceptable to strong loadings (β ≥ 0.50), several items showed relatively low loadings. Squared multiple correlations indicated acceptable explanatory power for the majority of items, supporting the adequacy of the final measurement model (Table 3).

**Table 2:** Model fit (N = 132).

	Absolute fit index			Incremental fitness index				Simple fit index	
	χ^2^	RMSEA	GFI	CFI	NFI	IFI	TLI	PNFI	PCFI
Initial model	328.53	0.07	0.84	0.89	0.79	0.89	0.87	0.67	0.76
Modified model	282.44	0.06	0.86	0.92	0.82	0.92	0.90	0.68	0.77

CFI = comparative fit index, GFI = goodness-of-fit index, IFI = incremental fit index, NFI = normed fit index, PCFI = parsimony comparative fit index, PNFI = parsimony normed fit index, RMSEA = root mean square error of approximation, TLI = Tucker–Lewis index.

**Table 3:** Standardized factor loadings and squared multiple correlations (*R*^2^) of the final CFA model.

Factor	Item	β (standardized loading)	*R* ^2^
Pain	Question 1	0.30	0.310
	Question 3	0.30	0.389
	Question 5	0.76	0.570
	Question 6	0.91	0.825
	Question 7	0.81	0.658
Miscellaneous	Question 2	0.39	0.383
	Question 4	0.40	0.362
	Question 13	0.37	0.305
Psychological destress	Question 8	0.38	0.306
	Question 9	0.63	0.392
	Question 10	0.55	0.303
	Question 11	0.74	0.548
	Question 12	0.42	0.372
Fear-avoidance	Question 14	0.57	0.327
	Question 15	0.53	0.383
	Question 16	0.76	0.572
Function	Question 17	0.83	0.685
	Question 18	0.87	0.752
	Question 19	0.92	0.842
	Question 20	0.79	0.622
	Question 21	0.65	0.427

CFA = confirmatory factor analysis.

### 3.3. Concurrent validity test

Concurrent validity of the Korean version of the OMPSQ was examined using Pearson correlation coefficients. The pain subscale of the OMPSQ showed a moderate positive correlation with the NRS (*r* = .50, *P* < .001). The function subscale of the OMPSQ was positively correlated with the SBST (*r* = .62, *P* < .001), and the psychological subscale was also correlated with the SBST (*r* = .60, *P* < .001). In addition, the fear-avoidance subscale of the OMPSQ demonstrated a positive correlation with the FABQ-K (*r* = .52, *P* < .001), supporting the concurrent validity of the instrument. According to Cohen criteria, these correlations represent large effect sizes, indicating clinically meaningful associations between the OMPSQ subscales and related instruments (Table 4).

**Table 4:** Correlation between the subscales of OMPSQ and each tool (N = 132).

Scale	OMPSQ subscale				Effect size
	Pain	Function	Psychological	Fear-avoidance	
	*r (P*)	*r (P*)	*r (P*)	*r (P*)	
NRS	0.50 (<.001)				Large
SBST		0.62 (<.001)	0.60 (<.001)		Large
FABQ-K				0.52 (<.001)	Large

Effect sizes were interpreted according to Cohen (1988) criteria (*r* ≥ 0.50 = large).

FABQ-K = Fear-Avoidance Belief Questionnaire-Korean, NRS = numerical rating scale, OMPSQ = Örebro Musculoskeletal Pain Screening Questionnaire, SBST = STarT Back Screening Tool.

### 3.4. Reliability test

The overall reliability of the Korean version of the OMPSQ was Cronbach α 0.84, confirming its high reliability. The Cronbach α values for each factor were pain 0.634, function 0.901, psychological distress 0.644, fear-avoidance 0.654, and miscellaneous 0.625, establishing the scale’s reliability.

## 4. Discussion

The OMPSQ is an effective tool for assessing psychosocial factors in patients with LBP to predict their risk of developing chronic pain and disabilities.^[14,15,31]^ However, the OMPSQ has not yet been translated or applied in Korea, and there is a need to develop a tool appropriate for Korean clinical settings and cultural characteristics.^[16,35]^ Therefore, this study aimed to translate the OMPSQ into Korean and provide a basis for effectively assessing psychosocial factors in Korean patients with LBP.

A CFA was conducted to verify the construct validity of the Korean version of the OMPSQ. CFA was used to assess whether the factor structure of an instrument fit the theoretical model assumed by the researcher, and various indicators were used to determine the fit. Generally, an RMSEA of 0.08 or less, CFI and GFI of at least 0.70, and a model fit of 0.90 or higher is considered good.^[38]^ The results of the CFA of the Korean version of the OMPSQ confirmed that it was a suitable model that retained the 5 subfactor structures of the original instrument. In the initial model analysis, some of the fit indices did not meet the criteria, but after adding covariance between error terms using MIs, fit indices such as RMSEA = 0.06, CFI = 0.92, and GFI = 0.86 were confirmed, indicating that the model fit was good. These results suggest that the Korean version of the OMPSQ can be translated and validated in Korean clinical settings while maintaining the same factor structure as the original instrument.

An MI was identified to improve model fit during CFA. In CFA, the modification index is an indicator that identifies poor fit caused by correlations between certain items that are not reflected in the model. Pairs of items with high values suggest they may measure the same factor but contain redundant information or lack independence between items. Adding inter-item covariance to the model can improve its fit and strengthen its structural validity.^[39]^ In the present study, MI analysis revealed high correlations between items 3 and 7 in the pain domain and 9, 10, 8, and 12 in the psychological domain. Based on this, we added inter-item covariance, which improved the model fit and confirmed a good model fit. These modifications improved the fit while maintaining the instrument’s measurement reliability.

These CFA findings may also be interpreted from a theoretical perspective. In particular, the elevated modification indices observed in the pain and psychological domains may be consistent with cultural tendencies in Confucian-influenced societies, where emotional expression is often restrained, and distress is internalized. Previous studies^[40,41]^ have reported that individuals in Asian cultural contexts tend to underreport pain and psychological distress. The strong inter-item correlations observed in the present study may therefore reflect such culturally patterned response tendencies. Future studies employing qualitative or mixed-method approaches are warranted to explore more deeply how cultural factors influence responses to psychosocial screening instruments.

The results of this study showed that the translation process of the Korean version of the OMPSQ has the potential to improve the structural validity of the instrument and adapt it to the Korean clinical setting. Furthermore, the effective handling of inter-item correlations within the same factor enhanced the internal consistency and reliability of the instrument and laid the foundation for a more accurate assessment of psychosocial factors in Korean patients with LBP.

The concurrent validity of the Korean version of the OMPSQ was verified, and each subscale showed significant correlations with related instruments. The “Pain” subscale of the OMPSQ correlated with the NRS at *r* = 0.50 (*P* < .001), the “Function” and “Psychological” subscales correlated with the SBST at *r* = 0.62 (*P* < .001) and *r* = 0.60 (*P* < .001), respectively, and the “Fear-Avoidance” subscale correlated with the FABQ-K at *r* = 0.52 (*P* < .001). These results confirm that the Korean version of the OMPSQ shows good concordance with comparable instruments for assessing pain and psychosocial factors and can be effectively used in Korean clinical settings. Furthermore, the correlation coefficients observed in our study (*r* = 0.47–0.62) were similar to those observed in Dutch studies^[42]^ (*r* = 0.38–0.64) and Brazilian Portuguese^[23]^ (*r* = 0.36–0.73) versions, supporting the consistency and reliability of the instrument.^[23,42]^ In particular, the Korean version of the OMPSQ is helpful in various clinical situations, such as chronic pain management and pain rehabilitation with psychological factors. However, the correlation coefficient of the fear-avoidance subscale was somewhat lower than that of the other language versions. In this study, participants had a mean duration of LBP of >34 months, whereas other studies focused on patients with <3 months.^[24,42]^ These differences in patient characteristics may explain the differences in the correlations of the fear-avoidance subscales. In addition, South Korea operates a mandatory National Health Insurance system, providing universal healthcare coverage to the population.^[43,44]^ Such a healthcare environment may alleviate concerns related to treatment accessibility and financial burden, potentially attenuating extreme scores on the FABQ-K that reflect work-related fear. From this perspective, the comparatively lower correlation observed in the fear-avoidance subscale in the present study may partly reflect contextual characteristics of the Korean healthcare system. Future studies should consider incorporating region-specific factors, such as healthcare access and insurance coverage, as covariates in analytical models to more precisely examine their influence on fear-avoidance beliefs.

In this study, the reliability of the Korean version of the OMPSQ was analyzed, and Cronbach α was 0.84, indicating high internal consistency. Generally, Cronbach α between 0.70 and 0.90 is considered good reliability,^[45]^ and the results of this study fully meet this requirement. The reliability values of this study are comparable to those of other language versions of the OMPSQ, with Cronbach α 0.81 for the Dutch version,^[42]^ 0.87 for the Brazilian version,^[23]^ and 0.84 for the Hong Kong version.^[24]^ These results demonstrate that the Korean version of the OMPSQ is as reliable as the other language versions and can be reliably used to assess psychosocial factors in Korean patients with LBP. However, despite the high Cronbach α observed in this study, test–retest reliability and potential floor or ceiling effects were not examined, which limits a comprehensive evaluation of the instrument’s longitudinal viability as a screening tool. Moreover, although a Cronbach α of 0.84 supports internal consistency, it should be interpreted with caution, as internal consistency alone does not substitute for predictive modeling or temporal stability. Future studies are therefore warranted to assess test–retest reliability over an interval of approximately 2 weeks, targeting an intraclass correlation coefficient >0.70, and to examine score distribution characteristics such as skewness. These extended analyses may also contribute to a more refined evaluation of the sensitivity of clinical cutoff scores.

Early screening of psychosocial factors plays a vital role in predicting the persistence and worsening of LBP and effectively managing it to improve patients’ quality of life. Clinical guidelines for chronic LBP also emphasize the importance of early identification of these factors to prevent long-term worsening of pain and provide appropriate treatment.^[1]^ In particular, screening patients at risk of long-term pain and disability and providing them with tailored treatment and management can contribute to reducing the social and economic burden of chronic pain.^[46]^ In this study, we developed the Korean version of the OMPSQ to screen for psychosocial factors related to chronic pain and disability in patients with LBP and validated its validity and reliability. The results showed that the Korean version of the OMPSQ had good construct validity, moderate concurrent validity, and high internal consistency, making it a suitable tool for assessing psychosocial factors in Korean clinical settings. This suggests that the Korean version of the OMPSQ can be a valuable tool for predicting the prognosis of patients with LBP in primary care and developing personalized treatment plans.

The limitations of this study are as follows:

- First, the study population was limited to patients with LBP who were visiting physiotherapy clinics in a specific region, which may limit the generalizability of the results. Therefore, further studies and validation with larger sample sizes are required.
- Second, data were collected using an online self-report survey, which may be subject to social desirability bias. However, the anonymous nature of the online survey may also be considered a strength, as it can facilitate more candid responses to psychosocial items.
- Third, digital data collection methods may pose challenges for older participants who are less familiar with technology, potentially leading to incomplete responses or reduced participation. In addition, online survey formats may increase acquiescence bias. Future research could address these limitations by employing mixed-mode data collection strategies, such as combining online and face-to-face assessments, to enhance response quality and inclusiveness.

Nevertheless, this study is significant because it developed a Korean version of the OMPSQ, validated its validity and reliability, and provided a basis for assessing psychosocial factors in Korean patients with LBP. Furthermore, the study suggests the practical feasibility of integrating the OMPSQ into initial LBP assessments in Korean clinical settings through app-based scoring, enabling a tiered referral pathway in which low-risk patients receive physiotherapy-focused management, while high-risk patients are referred for multidisciplinary interventions. This approach is expected to contribute to developing lower back pain management strategies that systematically incorporate psychosocial factors. Integrating the Korean OMPSQ into early clinical workflows may contribute to not only clinically effective but also cost-effective allocation of healthcare resources by directing intensive care towards patients at higher psychosocial risk. By aligning these implementation strategies with existing Korean pain management guidelines, the present study may be expected to extend beyond instrument validation to support real-world clinical application and inform policy-level integration of psychosocial screening in LBP management.

## 5. Conclusion

Collectively, our validation affirms the OMPSQ’s translatability, bridging Western-centric tools to East Asian contexts. The Korean version retained the original 5-factor structure and demonstrated acceptable psychometric properties, supporting its use for systematically assessing psychosocial factors in Korean patients with LBP. In addition, our multisite design (n = 163) and Beaton-compliant cross-cultural adaptation process ensure ecological validity. By providing a culturally adapted assessment tool suitable in primary physiotherapy contexts in South Korea, we found its potential to contribute to pain management and personalized treatment planning for patients with LBP. In particular, early assessment of psychosocial factors may support risk stratification and tailored care. Future studies should pursue targeted agendas, including prospective studies validating OMPSQ cutoffs in elderly cohorts and randomized controlled trials embedding the tool in stratified care. Furthermore, research should examine the validity of the OMPSQ across different cultural and clinical settings and diverse patient populations.

## Acknowledgments

The authors would like to express their sincere gratitude to the researchers who contributed to the translation and back-translation of the Örebro Musculoskeletal Pain Screening Questionnaire (OMPSQ) into Korean.

## Author contributions

**Conceptualization:** Kyoungrim Kang, Dong-Chul Moon, Sang-Hwa Lee.

**Data curation:** Kyo-Yeon Park.

**Formal analysis:** Kyoungrim Kang, Dong-Chul Moon, Sang-Hwa Lee, Kyo-Yeon Park.

**Investigation:** Kyoungrim Kang, Dong-Chul Moon, Sang-Hwa Lee, Kyo-Yeon Park.

**Writing – original draft:** Kyoungrim Kang, Dong-Chul Moon, Sang-Hwa Lee, Kyo-Yeon Park.

**Writing – review & editing:** Kyoungrim Kang, Dong-Chul Moon, Sang-Hwa Lee, Kyo-Yeon Park.
